# Short-active photoperiod gestation induces psychiatry-relevant behavior in healthy mice but a resiliency to such effects are seen in mice with reduced dopamine transporter expression

**DOI:** 10.1038/s41598-020-66873-2

**Published:** 2020-06-23

**Authors:** Molly A. Kwiatkowski, Zackary A. Cope, Maria L. Lavadia, Chuck J. A. van de Cappelle, Davide Dulcis, Jared W. Young

**Affiliations:** 1 Department of Psychiatry, University of California, San Diego, San Diego, USA; 2 Department of Medicine, Aging Institute, University of Pittsburgh, Pittsburgh, USA; 3 Division of Pharmacology, Utrecht Institute for Pharmaceutical Sciences, Utrecht University, Utrecht, The Netherlands; 4 Research Service, VA San Diego Healthcare System, San Diego, USA

**Keywords:** Neuroscience, Diseases

## Abstract

A higher incidence of multiple psychiatric disorders occurs in people born in late winter/early spring. Reduced light exposure/activity level impacts adult rodent behavior and neural mechanisms, yet few studies have investigated such light exposure on gestating fetuses. A dysfunctional dopamine system is implicated in most psychiatric disorders, and genetic polymorphisms reducing expression of the dopamine transporter (DAT) are associated with some conditions. Furthermore, adult mice with reduced DAT expression (DAT-HT) were hypersensitive to short active (SA; 19:5 L:D) photoperiod exposure versus their wildtype (WT) littermates. Effects of SA photoperiod exposure during gestation in these mice have not been examined. We confirmed adult females exhibit a heightened corticosterone response when in SA photoperiod. We then tested DAT-HT mice and WT littermates in psychiatry-relevant behavioral tests after SA or normal active (NA; 12:12 L:D) photoperiod exposure during gestation and early life. SA-born WT mice exhibited sensorimotor gating deficits (males), increased reward preference, less immobility, open arm avoidance (females), less motivation to obtain a reward, and reversal learning deficits, vs. NA-born WT mice. DAT-HT mice were largely resilient to these effects, however. Future studies will determine the mechanism(s) by which SA photoperiod exposure influences brain development to predispose toward emergence of psychiatry-relevant behaviors.

## Introduction

A higher incidence of multiple psychiatric disorders, including major depressive disorder (MDD), bipolar disorder (BD), and schizophrenia (SZ), occurs in people born in late winter/early spring^1,2^. Some factors arising from a winter gestation have been investigated to determine whether they drive outcomes that are relevant to psychiatric disorders, including maternal vitamin D deficiency and malnutrition (reviewed by Cope *et al*., 2016)^3^. To-date, little investigation has been made of reduced light exposure/activity levels, despite evidence they can impact adult behavior and neural mechanisms in rats and mice (see below). Altered light exposure during the perinatal period also induces lasting changes in the circadian clock of rodent offspring^4^. Relatively little investigation into the behavioral effects of altered perinatal photoperiod exposure has been conducted in such rodent offspring, although reduced immobility in the forced swim test (FST) has been reported (see Green *et al*., 2015)^5^. The question therefore remains as to what effect altered perinatal photoperiod exposure has on psychiatry-relevant behaviors in adults, that is behaviors observed in clinical psychiatric populations that can be observed in rodent models using cross-species translational tasks^6–13^.

In adult rodents, exposure to short active (SA; winter-like; 19:5 light:dark (L:D)) photoperiod increased stress-related hormones as well as psychiatry-relevant behavioral changes^14^. Specifically, after 7 days in a SA photoperiod, rats exhibited reduced time spent in the open arms of the elevated plus maze (EPM) and increased immobility in the FST, consistent with an anxiogenic- and depression-like profile. This profile coincided with neurotransmitter respecification occurring in the paraventricular nucleus of the hypothalamus which in a standard condition expressed tyrosine hydroxylase (TH), somatostatin (SST), and corticotropin-releasing factor (CRF), but in a SA condition expressed less TH, and more SST and CRF. A 14-day SA photoperiod exposure induced similar behavioral and neurotransmitter changes in mice, effects that were exaggerated in mice with reduced dopamine transporter (DAT) expression^15^. The effects of this stress-related response to winter-like-induced inactivity on gestation, however, have yet to be investigated.

While environmental factors such as winter gestation contribute to the development of psychiatric illnesses, genetic factors also play a role^16^. Many psychiatric disorders result from a complex interplay between environmental risk factors and an individual’s genetic background risk^17–20^, making the identification of genetic susceptibility/resiliency factors critical to understanding psychiatric disorder pathogenesis. Case-control, family cohort, and GWAS studies have repeatedly linked polymorphisms in *SLC6A3*, the gene encoding DAT, to multiple psychiatric conditions^21–36^. Polymorphisms in DAT—the main homeostatic mechanism for synaptic DA clearance—are associated with BD^23,35,37,38^, SZ^25–28^, and ADHD^21,22,29–32,39^. Reduced DAT levels (~40%) were observed in euthymic BD patients, and reduced DAT expression was confirmed in postmortem frontal cortices of BD patients^40^. Furthermore, DAT polymorphisms associated with psychiatric disease risk differentially modulate DAT gene promoter activity^41,42^. A subset of studies have not found an association between DAT and these disorders^43–46^; although a large number of studies support this association (above), and none determine potential interactive effects of season of birth. Such polymorphisms have been associated with reduced DAT expression and as indicated above, reduced DAT expression in mice induced a hypersensitivity to SA-photoperiod effects in adulthood^15^. Given that SA-photoperiod reduces levels of the precursor to dopamine, tyrosine hydroxylase^14,15^ there may be an interaction between genetic factors influencing DAT expression levels and photoperiod exposure on behavioral outcomes. Hence, reduced DAT expression could confer a similar sensitivity to photoperiod-induced behaviors during gestation and needs to be investigated.

To determine the effects of altered perinatal photoperiod on behavioral profiles in adulthood, we bred and raised mice in a SA vs. normal active (NA; 12:12 L:D) photoperiod. Additionally, we determined whether mice heterozygous for a DAT mutation that reduces DAT expression (DAT-HT) demonstrated an increased susceptibility to photoperiod-induced behavioral effects. We hypothesized that: 1) mice born/raised in SA photoperiod would exhibit psychiatry-relevant behavior vs. NA-born mice; and 2) DAT-HT mice would be hypersensitive to SA-induced behavioral changes compared to WT mice.

## Methods

### Dopamine transporter knockdown (DAT KD) line

A 7.5-kb *Hin*dIII fragment with the first two DAT gene exons was excised from a phage DNA isolated from a mouse 129 Sv/J genomic library, and a *Not*I—*Asc*I cassette was inserted to generate the targeting construct. The insertion of an extra 4-kb DNA sequence (tTA-neo-tetO) reduced DAT expression levels. W9.5 embryonic stem cells were electroporated with the linearized targeting construct and injected into C57BL6/J blastocysts to generate chimeras. One chimera was mated with 129 Sv/J females to generate heterozygous mutants (DAT-HT) on a 129 Sv/J background^47^. DAT-HT breeding pairs were sent to our laboratory from Columbia University and all subsequent mice resulted from a breeding colony in the vivarium at the University of California, San Diego (UCSD). Mice were bred until the DAT mutation was crossed over to a C57BL6/J background (>10 generations of backcrossing) and then maintained on this background. DAT-HT mice express ~50% of DAT compared to wildtype (WT) levels^15^.

### Animals for current experiments

**Figure 1:**
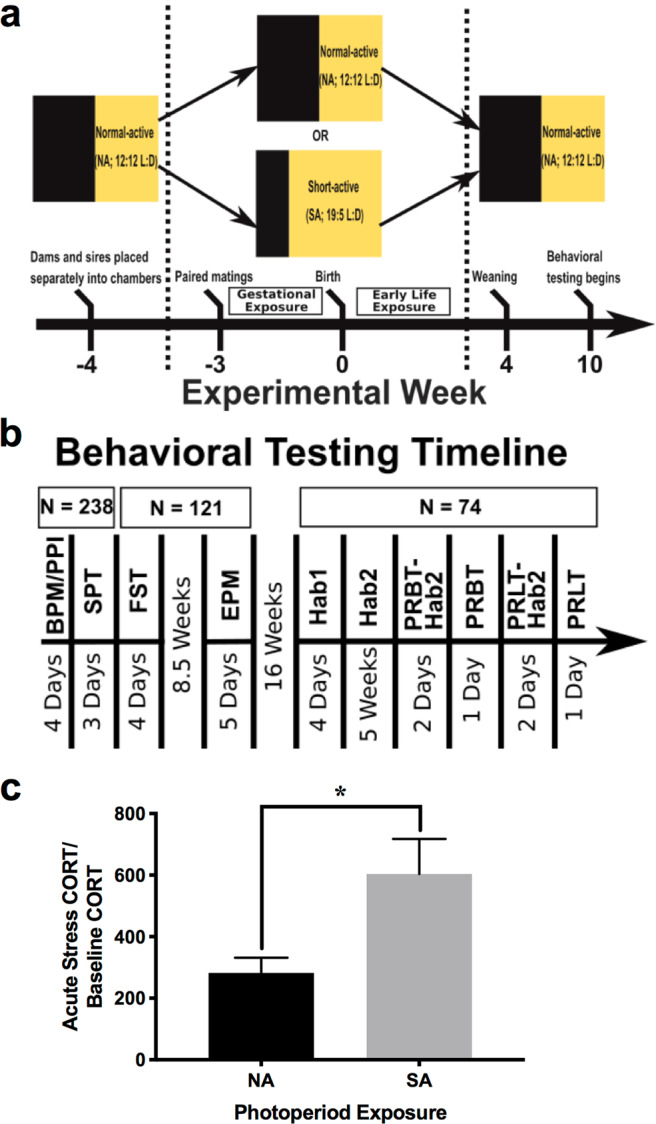
Schematic of experimental design and confirmation of short active photoperiod-induced stress response in adult females. (**a**) Schematic of the perinatal photoperiod exposure experimental timeline. Males heterozygous for a mutation in the dopamine transporter (DAT-HT) and female C57BL/6J (C57) breeders were placed separately into photoperiod chambers programmed to a normal active (NA; 12:12 light:dark (L:D)) cycle for 1 week. Following acclimation, breeding triads (2 female: 1 male) were formed at which point half the breeding pairs were moved to a short active (SA; 19:5 L:D) photoperiod. After 2 weeks, dams were singly housed and maintained in their respective photoperiods (NA or SA) throughout pregnancy into early life exposure (until weaning at P28). After weaning, offspring were housed in NA photoperiod (reversed L:D cycle) until behavioral testing began at 10–11 weeks old. (**b)** The behavioral testing timeline is described. All pups generated (N = 238) were tested in the behavioral pattern monitor (BPM), prepulse inhibition (PPI), and saccharin preference test (SPT). Subsequently, half (N = 121) were tested in the forced swim test (FST). After an 8.5-week washout period, they were tested in the elevated plus maze (EPM). After a 16-week period to minimize FST effects, a subset of the cohort (N = 74) were food deprived to 85% of free-feeding body weight, then trained in operant chambers and tested in the progressive ratio breakpoint task (PRBT) and probabilistic reversal learning task (PRLT). For Hab1, mice learned to associate magazine illumination with milkshake reward. For Hab2, mice were required to nose poke in any of the five illuminated holes to receive the milkshake reward. All mice were run on Hab2 for 2 days prior (PRBT-Hab2) to the single PRBT session, and 2 days prior (PRLT-Hab2) to the single PRLT session. (**c**) Confirmation of a SA photoperiod-induced stress response in females, wherein adult females exposed to SA photoperiod exhibited elevated plasma corticosterone (CORT) levels in response to acute restraint stress compared to NA-exposed female mice. Data presented as mean +S.E.M., *p < 0.05 where indicated.

Male (20–30 g) and female (15–25 g) DAT-HT and WT littermates were generated from DAT-HT (sire)/C57BL/6J (dam) pairings after being placed in custom photoperiod chambers (see Photoperiod Exposure Paradigm section below). Behavioral testing began at approximately 10 weeks old (see Fig. 1b for timeline). When not in the photoperiod chambers, all mice were group housed (3–4/cage) and were maintained on a reversed day-night light cycle (lights on 7:00 pm, off at 7:00 am) in a temperature-controlled vivarium. All behavioral tests occurred during the active (dark) phase of the cycle. Food and water were provided *ad libitum*. All procedures and tests were approved by the UCSD Institutional Animal Care and Use Committee. The UCSD animal vivarium meets all federal and state requirements for animal care and was approved by the American Association for Accreditation of Laboratory Animal Care.

### Plasma corticosterone assay

For confirmation of a SA-photoperiod stress-induced response at a hormonal level, adult female C57BL6/J mice were exposed to NA or SA photoperiod for 14 days, after which tail blood was collected for baseline plasma corticosterone (CORT) assessment. On day 15, tail blood was collected after a 2-hour acute restraint stress. Blood samples were analyzed for plasma CORT using a corticosterone rat/mouse ELISA kit (Rocky Mountain Diagnostics, Inc., Colorado Springs, CO, USA). A ratio of day 15 to day 14 plasma CORT levels was used for analyses.

### Photoperiod exposure paradigm

Male DAT-HT and female C57BL/6J breeders were placed into custom photoperiod chambers programmed to a NA (12:12 L:D) cycle prior to pairing in order to acclimate to the chambers (6 cages per chamber). Ten photoperiod chambers are individually ventilated and illuminated to 130 lumens (measured in the center of the chamber) by three horizontal, programmable white LED strips equally spaced on each wall. After a 7-day acclimation period, breeding triads (2 female: 1 male) were formed; half the breeding pairs remained in NA photoperiod, while half the breeding pairs were switched to SA photoperiod (19:5 L:D). Following a two-week pairing, dams were singly housed and maintained in their respective photoperiods (NA or SA) throughout pregnancy. The resulting 238 pups remained in either NA or SA photoperiod until weaning at P28. This photoperiod exposure was chosen to recreate the time period exposure of earlier research in this area^5^, as well as our work in adult photoperiod exposure inducing a stress-relevant response^14,15,48^. This time period was further supported given that neurodevelopmental timelines between mice and humans indicate that milestones in humans (e.g., peak brain growth spurts, 36–40 weeks) occur in mice post birth (postnatal days 7–10)^49^. Although not recreating the gradual change that occurs during human gestation, these conditions determine whether extreme photoperiod changes drive altered behavioral profiles in offspring. At P28, all pups were weaned by sex and genotype, moved into a standard vivarium room, and housed in NA photoperiod (12:12 L:D). For all subsequent behavioral testing, mice were maintained in NA photoperiod (see Fig. 1 for a schematic of experimental housing and testing timelines).

### Behavioral testing

To examine psychiatry-relevant behaviors induced by gestation/rearing in SA photoperiod, we assessed several domains commonly altered in patients with psychiatric disorders. Each test was performed as described below. The entire cohort of mice (N = 238) was tested in the behavioral pattern monitor (BPM), prepulse inhibition (PPI), and saccharin preference (SP) tests, whereas only half the cohort was used (n = 121) for the forced swim test (FST) and elevated plus maze (EPM). We used half the cohort for the latter two tests to minimize re-testing effects for other experiments (experiments not included here). An 8.5-week period elapsed between FST and EPM testing to minimize FST effects on EPM behavior (Fig. 1b). 16 weeks after EPM testing, a subset of these mice (n = 74; 8–10 per group) were trained in the progressive ratio breakpoint task (PRBT) and Probabilistic Reversal Learning Task (PRLT). See “Operant Training Phases” section below for details, and Fig. 1b for timeline of operant training and testing.

#### Prepulse inhibition (PPI): sensorimotor gating

Startle and PPI was tested using eight startle chambers (SR-LAB, San Diego Instruments, USA), each consisting of a Plexiglas cylinder (5 cm diameter) resting on a platform in a ventilated sound-attenuating box. Speakers located 33 cm above the cylinders produced all acoustic stimuli. Animal movements were transduced by piezoelectric accelerometers located underneath each cylinder, and data was stored and digitized by an interface and computer assembly. Mice were placed into startle chambers and underwent a 5-minute acclimation period prior to testing. Mice were exposed to a 65 dB background sound, as well as a light located on the chamber ceiling, continuously throughout the session.

Startle pulse duration was 40 ms, and prepulses were 20 ms in duration. The inter-trial interval (ITI) was on average 7 seconds (range: 3–12 s). The inter-stimulus interval (ISI) for prepulse trials was 100 ms (except for varying ISI trials below). Each session consisted of six blocks: (1) five 120 dB pulses, (2) prepulses (69, 73, 81 dB) preceding 100 dB pulses, (3) prepulses (69, 73, 81 dB) preceding 120 dB pulses, (4) varying ISI trials (73 dB prepulses preceding 120 dB pulses by 25, 50, 100, 200, and 500 ms, (5) startle trials (69, 73, 80, 90, 100, 110, 120 dB pulses), and (6) five 120 dB pulses. Startle responses to the 120 dB pulses from the first and sixth blocks were used to assess habituation across the session. PPI was calculated as a percentage for both the 100 dB and 120 dB pulses: %PPI = 100 – [(startle magnitude for prepulse+pulse/startle magnitude for pulse alone) × 100].

#### Behavioral pattern monitor (BPM): locomotor activity, exploratory behavior

Mice were tested in eight BPM chambers, each consisting of a 30.5 × 60 × 38 cm arena. Infrared beams designed to detect horizontal activity (e.g. number of transitions from one predefined region of the arena to another), vertical activity (e.g. rearing), and holepokes, tracked behavior across the 45-minute session. Behavioral data was acquired using Photobeam Activity System software (San Diego Instruments, USA). Custom programming was used to convert data into a format that could be analyzed using Biomedical Data Programs software (Statistical Solutions Inc., Saugus, MA). Detailed information regarding the chambers and testing methods have been previously published^50–53^. Mice were placed in the upper left corner of the chamber at the beginning of the session and could freely explore the arena. Primary outcomes included number of transitions, rearing, holepokes, and spatial d (a value quantifying the geometrical pattern of locomotor activity across the session, whereby values near 1 reflect straight line paths, values closer to 2 reflect a circumscribed path, and values near 1.5 reflect a meandering path). These measures were chosen given the difference between people with BD mania, acute schizophrenia episode, and healthy participants, and the ability to recreate such altered profiles in mouse models^7,54^.

#### Saccharin preference test (SPT): reward preference, hedonia-like behavior

Mice had 72-hour unrestricted access to both a normal water bottle and a water bottle filled with 1% saccharin solution in their home cage (3–4 mice/cage). Both bottles were weighed three times (once/day) by experimenters, and percent saccharin preference/cage was calculated as the primary outcome. All cage values were averaged within each group and compared to the other group averages.

#### Forced swim test (FST): despair-related behavior

Animals were placed into a 4.0L beaker filled with 2.5L of room temperature water. Animals were unable to touch the bottom of the beaker. The beaker was enclosed on all sides by a black box, and the experimenter observed behavior by video camera. The primary outcome variable, time spent immobile (seconds), was manually scored using ODLog software (Macropod Software) over the 5-minute session by people unblinded. Mice were judged “immobile” when not actively swimming. The first minute of data was subtracted to prevent obfuscation of perinatal photoperiod effects, as mice tend to be more active during the first portion of this test^55^.

#### Elevated plus maze (EPM): “anxiety-like” behavior, risk preference

Mice were placed in the center of the EPM at the beginning of the session. The EPM consisted of two open arms, two closed arms (Plexiglass walls covered with black paper), and an exposed center area. Mice could freely explore the maze for the entirety of the 5-minute session. Time spent in each open and closed arm, as well as in the center, was manually tracked using ODLog Software (Macropod Software). Primary outcomes included percent time spent in the open arms, percent time spent in the closed arms, and percent time spent in the center.

#### Operant training phases

Mice were initially trained in five-hole operant chambers (25 × 25 × 25 cm; Med Associates, St. Albans, USA). One week prior to initiation of Hab1 training, mice were food deprived to 85% of free-feeding body weight. During the first training phase (Hab1; 1 session/day), mice were conditioned to associate magazine illumination with the opportunity to collect 30 μl strawberry milkshake from the magazine. In the second training phase (Hab2; 1 session/day), mice were required to nose poke in any of the five illuminated holes to receive the reward (criterion: at least 70 responses for two consecutive days during 30-minute session). Once criterion was reached, mice moved into the testing phase.

#### Progressive ratio breakpoint task (PRBT): motivation to obtain reward

During a 60 min PRBT (tested on one day), mice had to make increasingly more holepokes into the central lit port in order to get a milkshake reward. The number of holepokes required to receive the reward increased in the following progression: 1, 2, 4, 7, 11, 16, 22, 29, 37, 46, 56, and 67 (see^56,57^). Mice had to respond three times at each ratio before moving on to the next ratio. If mice did not nose poke within 10 seconds, the chamber house light was illuminated for 4 seconds with all holes being inoperative until the next trial initiated. Primary outcome measure was breakpoint, defined as the last ratio completed before the end of the session. Secondary outcomes included total trials completed, mean reaction time, mean trial completion time, and mean response rate.

#### Probabilistic reversal learning task (PRLT): reversal learning

During a 60 min PRLT (tested on one day), mice were presented with two lit ports associated with different contingencies: the target port was associated with a high probability of reward (80%) and low probability of punishment (20%). The non-target port was associated with a low probability of reward (20%) and high probability of punishment (80%). After 8 consecutive responses in the target port, criterion was met, and the target port became the non-target port and vice versa (reversal). If mice did not nose poke within 10 seconds, the chamber house light was illuminated for 4 seconds with all holes being inoperative until the next trial initiated. Primary outcome measure was number of reversals completed in the 60 min session. Secondary outcomes analyzed for this task have been previously published by our lab^57^.

#### Operant training phase, PRBT, and PRLT data collection

Recording of responses in the operant training phases, as well as for PRBT and PRLT, was managed by a SmartCtrl Package 8-In/16-Out with additional interfacing by MED-PC for Windows (Med Associates, St. Albans, USA) using custom programming. Data was imported into Microsoft Excel using MED-PC to Excel (Med Associates, St. Albans, USA), and subsequently used for analysis.

### Statistical analyses

Primary outcomes from behavioral experiments were analyzed using ANOVA, with between-subjects factors of sex, genotype, and perinatal photoperiod exposure. Analyses were collapsed across sex where no main or interactive effect of sex was observed. For SPT, a within-subjects factor of time was included to assess differences in saccharin preference across the 3-day test period. For PPI, intra-block PPI measures (varying pulse intensities, ISI, or habituation), were added to the analysis as within-subjects factors. BPM data was analyzed across the entire 45-minute test session, as well as in 15-minute time bins (as a within-subjects factor). For the plasma corticosterone study, an unpaired t-test was used to assess for photoperiod effects on CORT levels. Data was analyzed using SPSS 24.0 (IBM Corp., Armonk, NY), except for BPM data, which was analyzed using Biomedical Data Programs software (Statistical Solutions Inc., Saugus, MA).

## Results

### SA-photoperiod effects on adult female plasma corticosterone levels

To determine whether the SA photoperiod drove a stress response in female mice, and hence had the potential to affect offspring, adult female mice were exposed to either NA or SA photoperiod for 14 days. Following this photoperiod exposure, SA-exposed adult female mice exhibited higher acute stress/baseline plasma corticosterone (CORT) ratios compared to NA-exposed females after a 2-hour acute restraint stress (t_(21)_ = 2.6, p < 0.05; Fig. 1c).

### Sensorimotor gating and startle: Prepulse inhibition (PPI)

**Figure 2:**
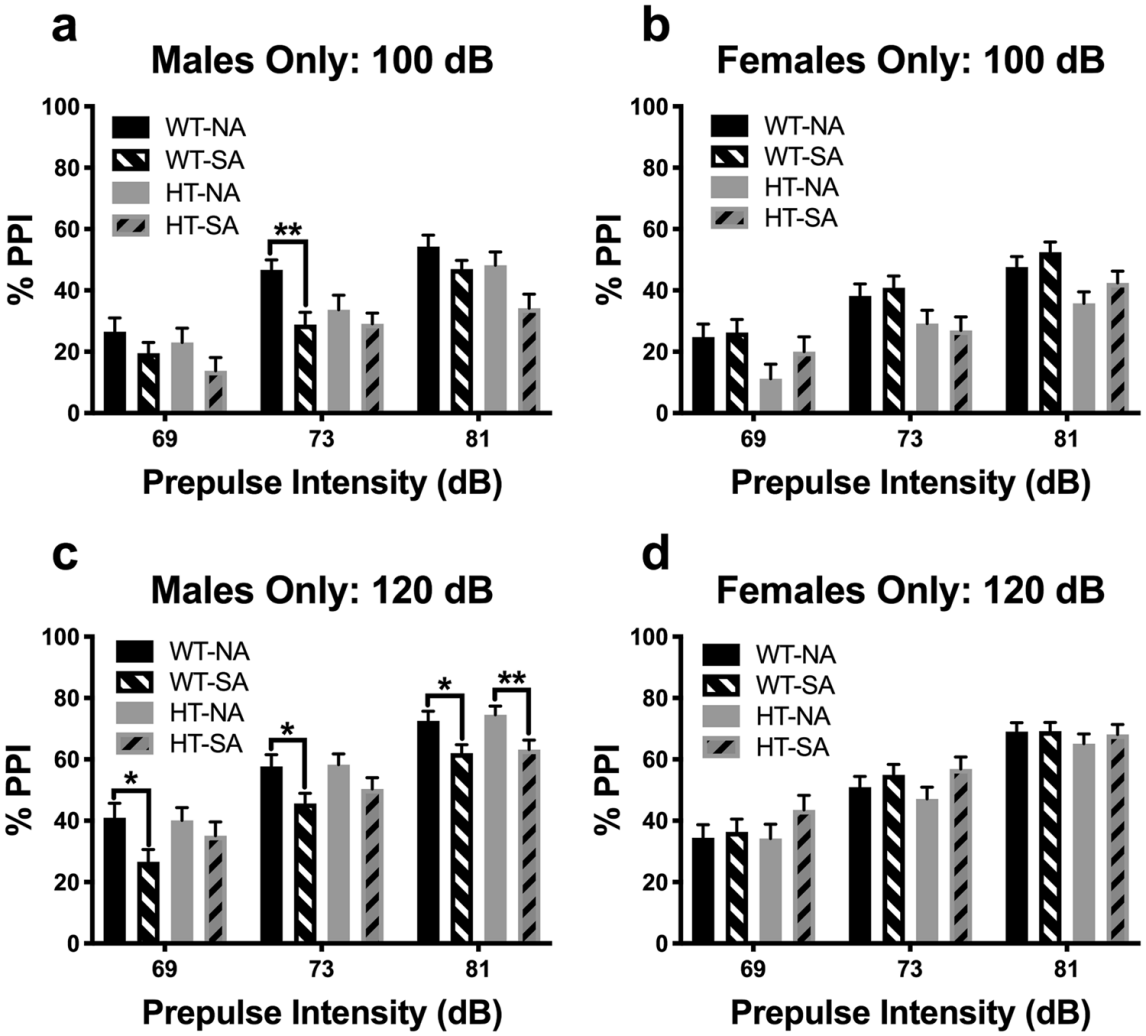
Short active (SA; 19:5 L:D) photoborn wildtype (WT) mice exhibit male-specific deficits in PPI. (**a**) Male SA photoperiod-born mice exhibited reduced prepulse inhibition (PPI) compared to normal active (NA; 12:12 L:D)-born male mice at a 100 dB startle pulse. This effect was specific to WT mice, not their dopamine transporter heterozygous (HT) littermates (**b**) This SA-photoborn-induced deficient PPI of WT mice was not seen in females, nor was there an effect in HT mice. Interestingly, female HT mice exhibited deficient PPI compared to female WT mice. **(c)** This pattern was recapitulated at 120 dB startle pulses wherein SA photoborn male WT mice exhibited deficient PPI, with little to no effect in HT mice (except at the 81 dB prepulse level). (**d**) Similarly, no interactions with or main effect of photoperiod were observed in female mice, either in WT or HT mice. Data presented as mean + S.E.M., *p < 0.05, **p < 0.01 where indicated.

Interactions with perinatal photoperiod were observed at both 100 dB and 120 dB. At 100 dB, a prepulse intensity x sex x genotype x perinatal photoperiod interaction was observed (F_(1.9,440)_ = 4.9, p < 0.01). Sex-specific analysis showed a prepulse intensity x genotype x perinatal photoperiod interaction in males, but not in females (males: F_(2,256)_ = 4.7, p < 0.05; females: F_(2,204)_ = 1.4, ns; Fig. 2a,b). Further restriction by genotype in males showed a prepulse intensity x perinatal photoperiod interaction in WT mice (F_(2,128)_ = 4.0, p < 0.05), with SA-born WT mice exhibiting reduced PPI compared to NA-born WT mice at the 73 dB prepulse level (p < 0.01). A main effect of photoperiod was also observed in male WT mice, with SA-born male WT mice exhibiting deficient PPI compared to NA-born male WT mice (F_(1,64)_ = 5.5, p < 0.05). There were no interactions with perinatal photoperiod observed in male DAT-HT mice; additionally, no main effect of perinatal photoperiod was observed (F_(1,64)_ = 2.9, ns). There was a main effect of genotype in female mice (F_(1,102)_ = 9.4, p < 0.01), with female DAT-HT mice exhibiting deficient PPI compared to female WT littermates. No significant interactions were seen in the pulse intensity or habituation curves at 100 dB.

At 120 dB, a sex x perinatal photoperiod interaction was observed (F_(1,230)_ = 9.8, p < 0.01). Sex-specific analysis showed a main effect of perinatal photoperiod in males only (F_(1,128)_ = 9.3, p < 0.01; Fig. 2c), with SA-born males exhibiting reduced PPI compared to NA-born males. At each prepulse intensity, SA-born male WT mice exhibited deficient PPI compared to NA-born male WT littermates; this difference was only significant at the highest prepulse intensity in SA-born vs. NA-born male DAT-HT mice. No interactions or main effects were observed in female mice, except for a main effect of prepulse intensity (Fig. 2d). No sex x perinatal photoperiod interactions were observed in the pulse intensity or habituation curves at 120 dB.

### Exploratory behavior: Behavioral pattern monitor (BPM)

**Figure 3:**
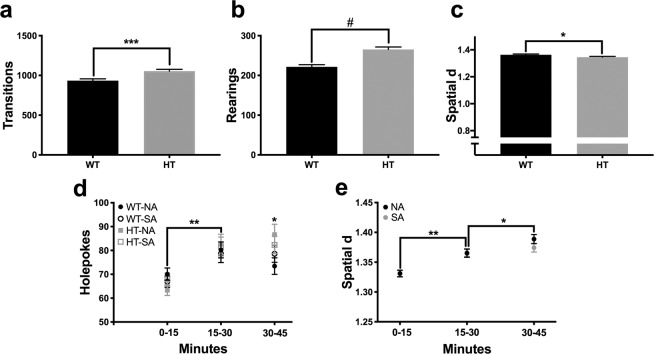
Short active (SA; 19:5 L:D) photoperiod did not induce changes in locomotor activity or exploratory behavior, as measured in the behavioral pattern monitor (BPM). (**a**) Photoperiod birth neither affected, nor interacted with genotype to affect transitions, (**b**) rearing, or (**c**) spatial d. Dopamine transporter heterozygous (HT) mice exhibited more transitions, rearing, and lower spatial d (more straight-line movement) than their wildtype (WT) littermates, however. (**d**) Across 15-min time bins, all groups exhibited increased holepoking from the first to the second time bin, while normal active (NA; 12:12 L:D) photoborn HT mice holepoked more than their NA-born WT littermates in the final time bin. (**e**) Both NA- and SA-born mice exhibited an increased spatial d between the first and second time bin, while NA-born mice exhibited further increased spatial d in the final time bin. Data presented as mean ±S.E.M., *p < 0.05, **p < 0.01, ***p < 0.001, ^#^p < 0.0001 where indicated.

When data were analyzed in a single 45-minute time bin, no interactions with or main effects of perinatal photoperiod were observed in any BPM primary outcome measures (number of transitions, rearing, holepokes, or spatial d). A main effect of genotype was observed for number of transitions (F_(1,230)_ = 13.9, p < 0.001; Fig. 3a), rearing (F_(1,230)_ = 28.0, p < 0.0001; Fig. 3b), and spatial d (F_(1,230)_ = 5.1, p < 0.05; Fig. 3c). DAT-HT mice exhibited increases in number of transitions and rearings, as well as a reduction in spatial d, compared to WT littermates.

When data were analyzed in 15-minute time bins, a time x genotype x perinatal photoperiod interaction was observed for holepokes (F_(2,460)_ = 3.1, p < 0.05; Fig. 3d). All groups, regardless of genotype or perinatal photoperiod exposure, exhibited a significant increase in holepokes between the first and second 15-minute time bin (NA-born WT: t_(112)_ = 2.4, p < 0.05; NA-born DAT-HT: t_(116)_ = 4.0, p < 0.01; SA-born WT: t_(134)_ = 2.7, p < 0.01; SA-born DAT-HT: t_(104)_ = 2.9, p < 0.01). When examining for inter-group differences at each time point, no significant differences were observed during the first or second 15-minute time bin. During the final 15-minute time bin, NA-born DAT-HT mice completed significantly more holepokes compared to NA-born WT mice (t_(114)_ = 2.3, p < 0.05).

A time x perinatal photoperiod interaction for spatial d was observed when data were analyzed in 15-minute time bins (F_(2,240)_ = 5.3, p < 0.01; Fig. 3e). Both NA-born and SA-born mice exhibited an increased spatial d between the first and second 15-minute time bin (NA: t_(230)_ = 3.9, p < 0.01; SA: t_(240)_ = 4.2, p < 0.0001). A further increase in spatial d was observed between the second and third 15-minute time bin in NA-born mice (t_(230)_ = 2.3, p < 0.05); this effect was not observed in SA-born mice (t_(240)_ = 0.8).

### Reward sensitivity: Saccharin preference test (SPT)

**Figure 4:**
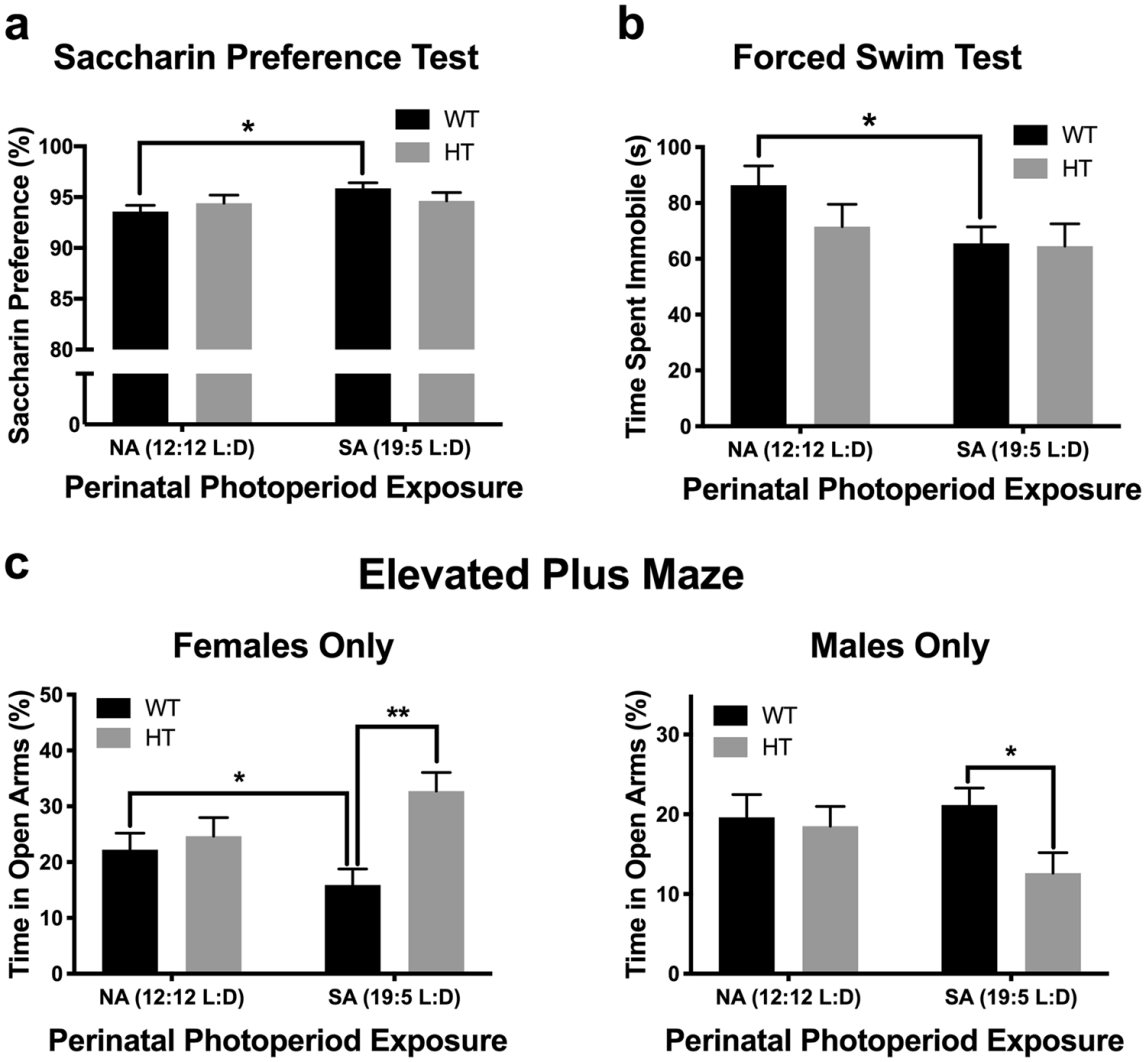
Short active (SA; 19:5 L:D) photoperiod born-induced changes on ethologically-relevant behaviors. **(a)** SA-born wildtype (WT) mice exhibited increased saccharin preference compared to normal active (NA; 12:12 L:D)-born WT mice, while dopamine transporter heterozygous (HT) mice were resilient to this effect. **(b)** SA-born WT mice exhibited decreased immobility compared to NA-born WT mice, with HT mice again unaffected. **(c)** In females (left), SA-born WT mice spent significantly less time in open arms compared to female NA-born WT mice, while SA-born female HT mice spent significantly more time in open arms compared to SA-born female WT mice. In males (right), male SA-born HT mice spent less time in open arms compared to male SA-born WT mice. Data presented as mean +S.E.M., *p < 0.05, **p < 0.01 where indicated.

When tested as adults, mice born and raised in SA photoperiod tended to exhibit increased saccharin preference when compared to NA-born mice (F_(1,51)_ = 3.2, p = 0.08). No interactions with sex or genotype were observed in the overall analysis. Given our a priori hypothesis of genotype-specific effects, we performed genotype-specific analyses of SP. SA-born WT mice exhibited increased saccharin preference compared to NA-born WT mice (F_(1,26)_ = 7.8, p < 0.05; Fig. 4a), whereas there were no photoperiod effects in DAT-HT mice (F_(1,25)_ = 0.0, ns; Fig. 4a).

### Behavioral despair: Forced swim test (FST)

When tested as adults, mice born and reared in SA photoperiod exhibited a trend toward decreased immobility in the FST, regardless of genotype, when compared to NA-born mice (F_(1,113)_ = 3.8, p = 0.06). A main effect of sex was also observed, with males spending more time immobile compared to females (F_(1,113)_ = 16.7, p < 0.001). Given our a priori hypothesis that these effects would be genotype-specific, we investigated photoperiod effects in each genotype. In WT mice, there was a main effect of photoperiod (F_(1,61)_ = 5.2, p < 0.05; Fig. 4b), where SA-born WT mice exhibited decreased immobility compared to NA-born WT mice. In contrast, no photoperiod effect was observed in DAT-HT mice (F_(1,52)_ = 0.4, ns; Fig. 4b).

### Risk preference/anxiety assessment: Elevated plus maze (EPM)

A sex x genotype x perinatal photoperiod interaction for percent time spent in open arms was observed in the EPM (F_(1,112)_ = 6.0, p < 0.05). When males and females were analyzed separately, no interactions with or main effects of perinatal photoperiod were observed. In females only, a trend toward a genotype x perinatal photoperiod interaction was observed (F_(1,51)_ = 3.7, p = 0.06). Female SA-born WT mice spent significantly less time in open arms compared to female NA-born WT mice (t_(29)_ = 2.1, p < 0.05; Fig. 4c). SA-born female DAT-HT mice spent significantly more time in open arms compared to SA-born female WT mice (t_(26)_ = 2.9, p < 0.01; Fig. 4c). A main effect of genotype was also seen in female mice, with DAT-HT mice spending more time in open arms compared to WT mice (F_(1,51)_ = 6.5, p < 0.05). In male mice, SA-born DAT-HT mice spent less time in open arms compared to SA-born WT mice (F_(1,36)_ = 6.9, p < 0.05; Fig. 4c).

### Effort valuation: Progressive ratio breakpoint task (PRBT)

**Figure 5:**
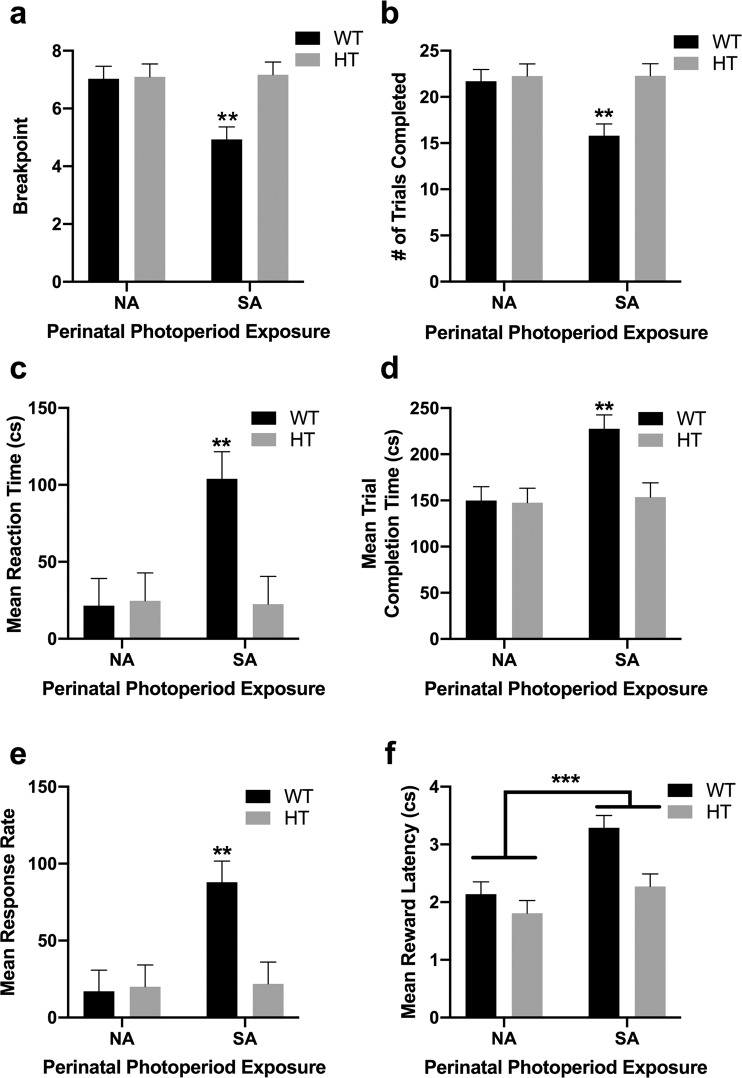
Short active (SA; 19:5 L:D) photoperiod induced a reduction in willingness to work for a reward in wildtype (WT) mice. (**a**) SA-born WT mice exhibited a reduced breakpoint, (**b**) thus completed fewer trials, (**c**) exhibited longer reaction times, and (**d**) took longer to complete trials compared to all other groups. (**e**) SA-born WT mice had increased response rates, compared to all other groups. (**f**) Importantly, SA-born mice took longer to collect earned rewards than NA-born mice, largely driven by SA-born WT mice. No main effect of dopamine transporter heterozygous (HT) genotype, or effect of photoperiod on HT mice, was observed for any measure. Data presented as mean +S.E.M., **p < 0.01, ***p < 0.001 where indicated.

During Hab2 training, there was a sex x genotype x perinatal photoperiod interaction (F_(1,66)_ = 5.9, p < 0.05). SA-born male WT mice took significantly more sessions to meet criterion than all other groups (p < 0.0001 vs. female DAT-HT NA; p < 0.001 vs. female WT NA, female WT SA, male DAT-HT SA; p < 0.01 vs. female DAT-HT SA, male WT NA, male DAT-HT NA). In the PRBT, a genotype x perinatal photoperiod interaction was observed for breakpoint (F_(1,66)_ = 6.1, p < 0.05; Fig. 5a). SA-born WT mice exhibited a reduced breakpoint vs. NA-born WT mice (F_(1,36)_ = 12.6, p < 0.01) as well as vs. SA-born DAT-HT mice (F_(1,35)_ = 10.5, p < 0.01). Genotype x perinatal photoperiod interactions were also observed for most secondary outcome measures. SA-born WT mice completed fewer trials (F_(1,66)_ = 5.3, p < 0.05; Fig. 5b), had slower reaction times (F_(1,66)_ = 5.6, p < 0.05; Fig. 5c), took longer to complete each trial (F_(1,66)_ = 5.5, p < 0.05; Fig. 5d), and exhibited increased mean response rates (F_(1,66)_ = 6.1, p < 0.05; Fig. 5e) compared to all other groups. A main effect of perinatal photoperiod was also observed on mean reward latency, with SA-born mice taking longer to retrieve earned rewards than NA-born mice (F_(1,66)_ = 13.8, p < 0.001; Fig. 5f).

### Reversal learning: Probabilistic reversal learning task (PRLT)

**Figure 6:**
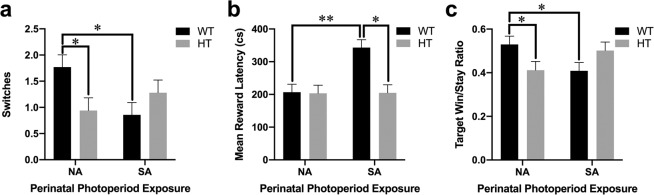
Short active (SA; 19:5 L:D) photoperiod induced impaired probabilistic learning in wildtype (WT) mice. (**a**) SA-born WT mice completed fewer switches compared to normal active (NA; 12:12 L:D)-born WT mice. NA-born dopamine transporter heterozygous (HT) mice completed fewer switches compared to NA-born WT mice. (**b**) SA-born WT mice took longer to collect earned rewards than NA-born WT mice and SA-born DAT-HT mice. (**c**) SA-born WT mice were less likely to choose the target after being rewarded from choosing the target in the previous trial (target win/stay ratio) compared to NA-born WT mice. NA-born DAT-HT mice also exhibited a reduced target win/stay ratio compared to NA-born WT mice. Data presented as mean +S.E.M., *p < 0.05, **p < 0.01 where indicated.

A genotype x perinatal photoperiod interaction was observed for number of switches completed during the PRLT session (F_(1,66)_ = 6.9, p < 0.05; Fig. 6a). SA-born WT mice completed fewer switches compared to NA-born WT mice (F_(1,36)_ = 5.9, p < 0.05; Fig. 6a). NA-born DAT-HT mice completed fewer switches compared to NA-born WT mice (F_(1,35)_ = 5.0, p < 0.05; Fig. 6a). For secondary outcome measures, a genotype x perinatal photoperiod interaction was observed for mean reward latency (F_(1,66)_ = 7.6, p < 0.01; Fig. 6b) and target win-stay ratio (F_(1,66)_ = 7.3, p < 0.01; Fig. 6c). Target win-stay ratio represents the probability of choosing the target port after being rewarded from choosing the target port in the previous trial. SA-born WT mice exhibited an increased latency to collect rewards after being rewarded compared to both NA-born WT mice (F_(1,36)_ = 7.8, p < 0.01) and SA-born DAT-HT mice (F_(1,35)_ = 7.4, p < 0.05). SA-born WT mice exhibited a reduced target win-stay ratio compared to NA-born WT mice (F_(1,36)_ = 5.6, p < 0.05). NA-born DAT-HT mice also showed a reduced target win-stay ratio relative to NA-born WT mice (F_(1,35)_ = 5.8, p < 0.05).

## Discussion

Exposure to short active (SA; 19:5 L:D) photoperiod increased corticosterone levels in adult female mice. Exposure to this SA photoperiod during pregnancy and early life induced psychiatry-relevant behavioral profiles in WT mice. Specifically, SA-born WT mice exhibited reduced PPI (males only), increased saccharin preference, reduced FST immobility, less time spent in open arms of EPM (females only), less motivation to obtain a reward (breakpoint; PRBT), and reversal learning deficits (switches; PRLT), compared to NA-born WT mice (see Table 1 for summary of behavioral results). No SA photoperiod effects were observed in the BPM, suggesting perinatal SA photoperiod exposure does not alter locomotor activity or exploratory behavior. Surprisingly, mice with reduced DAT expression (DAT-HT mice) that were hypersensitive to SA photoperiod effects in adulthood^15^, were largely resistant to the effects of gestational SA photoperiod exposure (with the exception of significant PPI deficits observed at the highest prepulse level, 81 dB; Table 1). Thus, recreating a winter-like SA photoperiod exposure in mice during gestation resulted in psychiatry-relevant behavioral abnormalities in adulthood, for which mice with reduced DAT expression were resilient.

**Table 1:** Behavioral effects of short active (SA; 19:5 L:D) photoperiod exposure during gestation and early life in mice heterozygous for a mutation in the dopamine transporter (DAT-HT) and wildtype (WT) littermates. PPI = prepulse inhibition; BPM = behavioral pattern monitor; SPT = saccharin preference test; FST = forced swim test; EPM = elevated plus maze; PRBT = progressive ratio breakpoint task; PRLT = probabilistic reversal learning task.

Behavior (Test)	Potential Relevance to Human Condition	Effect in WT (vs. NA-born WT)	Effect in DAT-HT (vs. NA-born DAT-HT)
Sensorimotor gating (PPI)	Deficits seen in patients with schizophrenia (SZ)^58,59^, obsessive-compulsive disorder (OCD)^60,61^, autism spectrium disorder (ASD) in children/ adolescents^62^	100 & 120 dB: **↓** PPI (males only)	100 dB: no effect 120 dB: **↓** PPI (males only; 81 dB prepulse level only)
Locomotor activity/ Exploration (BPM)	Hyperlocomotion, hyperexploration, and altered motor activity patterns seen in patients with mania^51,97,98^	**None**	**None**
Reward preference, Hedonia (SPT)	Used in rodent models as a measure of anhedonia^65^; anhedonia cardinal feature of major depressive disorder (MDD)^64^	**↑** SP	**None**
Despair-related behavior (FST)	Commonly used in rodent models as metric for depression-like behaviors seen in humans (e.g. despair)^69,70^	**↓** immobility	**None**
“Anxiety-like” behavior, risk preference (EPM)	Used in animal models to measure anxiety behavior seen in humans^99,100^	**↓** time spent in open arms (females only)	**None**
Motivation to obtain reward (PRBT)	Decreased breakpoint in patients with SZ^75,101^, patients with MDD less willing to expend effort to obtain reward^102^	**↓** breakpoint (less motivated)	**None**
Reversal learning (PRLT)	Impaired reversal learning in SZ^76,78^, OCD^103^	**↓** reversal learning	**None**

The behavioral tests in the current study were selected to span a broad array of psychiatry-relevant behaviors consistent with those observed in patient populations. PPI deficits (reflecting sensorimotor gating deficits) have been observed in patients with SZ^58,59^, obsessive-compulsive disorder (OCD)^60,61^, and children/adolescents with ASD^62^. Cortico-striato-pallidopontine (CSPP) circuitry regulates PPI, and has been implicated in the pathology of multiple psychiatric disorders^63^. SA perinatal photoperiod exposure may therefore influence CSPP circuitry (given the observed SA-induced PPI deficits), reflecting one way altered photoperiod exposure near birth might contribute to emergence of psychiatry-relevant behaviors in adulthood. Two of the tests included in this study (SPT, FST) are commonly used in animals to provide insight into depression-relevant behaviors. Given that anhedonia is a cardinal feature of MDD^64^, it is thought that the SPT measures such behavior in rodents^65^, despite unaltered SPT in people with depression relative to healthy participants^66–68^. Time spent immobile in the FST is a biological readout of altered behavior and has been associated with depression-relevant behaviors (e.g. behavioral despair)^69,70^. In both tests, SA-born WT mice exhibited the opposite of expectations of ‘anhedonic’-like profile, exhibiting increased saccharin preference and decreased FST immobility. While the relationship of these findings to aspects of depression remain unclear, the photoperiod gestation challenge affected behavior in these tests. The mechanism driving these effects remains unclear, but it is important to note that a similar photoperiod manipulation resulted in reduced FST immobility in a previous study^5^; this consistency across photoperiod studies (albeit with minor mouse background and 1 hour light change differences), from different laboratories supports the validity of these outcome measures. That study suggested the SA-born-induced reduction in immobility may have been as a result of a hyper-serotonergic dorsal raphe nuclei, but the dopaminergic system may also play a role in this effect given that mice with reduced DAT expression were resistant to this SA-born effect.

Although FST immobility has commonly been used as a metric for depression-relevant behaviors (e.g. despair), this interpretation may not accurately reflect what the behavior represents. Some suggest immobility is an adaptive learned response and reflects a coping strategy to the FST stressor^71^. Others argue that reduced immobility may represent an anxiogenic behavioral profile, rather than an ‘antidepressant’ effect^72^. Therefore, the reduced immobility seen in SA-born mice could be an anxiogenic state or a maladaptive response to stress. An anxiogenic state is further supported by SA-born WT mice (females) spending less time in the open arms of the EPM. However, it is important to note that prior FST exposure has been shown to induce an anxiogenic behavioral profile in the EPM (in male mice)^73^. As a result, we cannot rule out the possibility that FST testing influenced EPM results. Reduced breakpoint (PRBT) and impaired probabilistic learning (PRLT) are outcomes viewed as more proximate to amotivated behavior in SZ^74–76^ and depression^77^. Furthermore, these tests are more translationally relevant, as versions of these tasks have been implemented in both rodents and human populations. Reduced breakpoint from a PRBT predicts 24% of the variance of global cognitive functioning in patients with SZ, with lower breakpoints associated with greater severity of certain negative symptoms (anhedonia, avolition)^75^. Impaired PRLT has also been observed in patients with SZ^78^. Given that FST immobility more than likely does not serve as an accurate measure of motivated behavior, the decreased FST immobility and reduced breakpoint observed in SA-born mice in the current study are not necessarily contradictory. That slowed latencies to collect rewards were observed in SA-born WT mice in both the PRBT and PRLT indicate a reduced motivation to exert effort to collect rewards. The lack of difference of overall activity levels in an exploratory chamber (BPM), suggests this amotivated behavior is specific to the collection of reward. Given the reduced win-stay behavior of SA-born WT mice, these deficits may also arise from impaired reward processing. Future studies examining effort-related choice will likely be useful in determining the impact of this manipulation on motivated behaviors.

Some sex-specific results were observed in the current study. SA-born males (particularly WT males) exhibited reduced PPI, whereas SA-born female WT mice exhibited an anxiogenic profile in the EPM. These findings are consistent with other reports of sex-specific changes as a result of perinatal stress exposure, and how these differences might contribute to subsequent health outcomes of offspring^79^. One study showed greater prenatal maternal stressor sensitivity in male offspring for subsequent development of SZ spectrum disorders^80^. Studies have suggested that female fetuses are more flexible in adapting to adverse intrauterine conditions compared to male fetuses, but that this adaptability comes with a tradeoff of increased susceptibility to anxiety/depression^81^. This female-specific vulnerability to developing depression-like behavior has also been shown in response to early life stress exposure using the limited bedding model to induce fragmented maternal care^82^. Epidemiological studies have observed a winter/spring birth excess in people with depression^1,83–85^, and one study reported a higher excess in women (peak excess of 31% in women born in March compared to women in general population comparison group)^86^. Other studies report male vulnerability to this seasonality effect^87^, while some report no association between birth season and depression for either sex^88^. Studies to date have been limited, and more work is required to understand seasonality of birth effects on risk for affective disorder development. The sexually dimorphic results we observed fit within the existing prenatal stress vulnerability literature. Given the observation of limited sex-specific effects in the DAT HT mice – which were largely resilient to SA photoperiod birth effects – strengthen the implication that the sex-specific effects of such birth on WT mice fit into the literature. Future work should examine whether altered perinatal photoperiod exposure induces changes in maternal stress hormones (e.g. plasma corticosterone), which could be conferred to developing fetuses in a sex-specific manner.

Although we predicted that DAT-HT mice would be hypersensitive to SA-induced behavioral changes given their hypersensitivity in adulthood, they were resilient to photoperiod manipulation effects compared to their WT littermates. Investigations into the mechanisms underlying vulnerability (and, conversely, resiliency) to stress have recently become more numerous. Glucocorticoid administration during late gestation influences dopaminergic neural circuits, inducing an increased ratio of apoptotic to proliferative cells in the ventral tegmental area and subsequent reduction in dopaminergic input to the nucleus accumbens^89^. While the effects of early life stress on dopaminergic circuitry are complex (reviewed here^90^), the DAT mutation may protect against SA-induced behavioral changes by somehow counteracting photoperiod effects on developing dopaminergic pathways. The mechanism(s) underlying the observed resilience in DAT-HT mice remains unknown, however, and future work will determine how resilience is conferred.

In summary, SA perinatal photoperiod induces psychiatry-relevant behavioral profiles later in adulthood in mice, with vulnerability observed in WT mice. It is recognized that this photoperiod effect was consistent throughout but abrupt in its change, unlike changing light exposure in humans. Future studies will determine the mechanism(s) by which SA photoperiod exposure influences brain development to predispose toward emergence of psychiatry-relevant behaviors, as well as attempt to recreate gradual change effects on adult offspring. First, identifying the window of SA photoperiod exposure necessary to induce these behavioral effects will be critical to narrowing in on potential mechanisms (gestation vs. early life vs. a combination of the two time periods). If SA photoperiod exposure during gestation only is capable of inducing behavioral changes in offspring, stress-related changes in placental structure/gene expression might be contributing mechanisms. The placenta plays an active role in fetal brain development^91^, and maternal environment perturbations (e.g. hormone levels, nutritional state) are transmitted to the fetus via changes in placental function^92^. In adult rodents, SA photoperiod exposure results in elevated plasma corticosterone^14^, and exposure to elevated levels of maternal corticosterone affects placental growth and gene expression^93^. These gene expression changes may alter the transplacental signals received by the developing fetus, and by extension, the developing brain. Furthermore, maternal signals are capable of affecting long-term gene expression changes in offspring via induction of epigenetic mechanisms^94^. If behavioral changes occur after SA exposure during early life (post-gestational), it may point toward other mechanisms. Retinal projections reach the suprachiasmatic nucleus (central clock of the circadian system) shortly after birth^95^. Perinatal photoperiod-induced changes in retinal function^96^ and circadian rhythms^4^ have been previously reported, and may contribute to observed behavioral changes in offspring by influencing physiological crosstalk with the stress system^95^, thereby altering stress responses in adulthood. Many other potential mechanisms exist, and likely it is a combination of pre- and postnatal influences converging to produce SA-induced behavioral profiles. The interaction between environmental factors and developing neural circuitry is a critical part of the etiology of many psychiatric disorders; a better understanding of these complex processes will allow for the development of more effective therapeutics.
